# Short-term Outcomes in Pediatric Patients Managed with Peripheral Nerve Blockade for Arthroscopic Anterior Cruciate Ligament Reconstruction and/or Meniscus Surgeries

**DOI:** 10.7759/cureus.2852

**Published:** 2018-06-21

**Authors:** Alexander J Adams, Wallis T Muhly, Harshad G Gurnaney, Joy C Kerr, Lawrence Wells

**Affiliations:** 1 Orthopaedic Surgery, The Children's Hospital of Philadelphia; 2 Anesthesiology/Critical Care, Children's Hospital of Philadelphia, Philadelphia, USA; 3 Anesthesiology, The Children's Hospital of Philadelphia, Philadelphia, USA; 4 Orthopaedics, The Children's Hospital of Philadelphia, Philadelphia, USA

**Keywords:** peripheral nerve blockade, pediatric knee arthroscopy, anterior cruciate ligament, meniscus, short-term outcomes, analgesia, post-discharge

## Abstract

Introduction

Peripheral nerve blockade (PNB) can be a useful component of a multimodal analgesia approach in managing pain after knee arthroscopy. However, the impact of PNB and short-term recovery in pediatric patients, particularly adolescents, who underwent knee arthroscopy for anterior cruciate ligament (ACL) reconstruction and/or meniscus surgery (repair or resection) has not been well characterized. This prospective study presents observational data on short-term patient outcomes and side effects for 72 hours following discharging home of pediatric patients who underwent arthroscopic ACL and/or meniscus procedures with PNB.

Methods

This is a single-center, single-surgeon prospective observational study conducted over a three-year period. We characterized 72-hour postoperative outcomes including pain scores, return of sensation to the affected limb, analgesic use [nonsteroidal anti-inflammatory drugs (NSAIDs) and opioids], readmission rate, and activities of daily living (ADL) via telephone survey. In addition, retrospective chart review was conducted to obtain perioperative and anesthesia details. Results for surgery groups were analyzed using descriptive and Pearson correlations using the SPSS version 24 (IBM Corp. Released 2016. IBM SPSS Statistics for Mac, Version 24.0. Armonk, NY, USA).

Results

We collected data on 47 patients undergoing ACL reconstruction with or without meniscus surgery (18/47, 38.3%) or meniscus surgery only (29/47, 61.7%). At 72 hours postsurgery, there were no readmissions or complications related to pain. Median-reported pain scores were 2.5 and 5.0 for the ACL and meniscus groups, respectively. A majority of patients continued to require opioids (45/47, 95.7%) and NSAIDs (46/47, 97.9%) at 72 hours postsurgery, but the number of daily opioid doses taken decreased with each day postoperatively. Over 93% of the patients could ambulate and shower at 72 hours postsurgery.

Conclusions

Regional nerve block appears to be an effective and safe analgesic strategy for pediatric arthroscopic ACL and meniscus procedures, with no short-term complications or readmissions related to pain in our cohort. Future prospective investigation is needed to characterize long-term pain outcomes in this surgical population.

## Introduction

Effective pain control following a surgery is critical, and it is increasingly clear that employing a multimodal approach for acute pain management can improve patient outcomes [1-2]. Multimodal analgesia refers to the use of multiple methods [nonsteroidal anti-inflammatory drugs (NSAIDs), local infiltration, peripheral nerve blocks when possible] in an effort to improve pain relief and minimize opioid consumption. Recently, the American Society of Anesthesiologists (ASA) and the American Pain Society (APS) released comprehensive evidence-based guidelines strongly recommending the use of multimodal analgesia in the wake of the prescription opioid crisis [3].

In orthopedic surgery, peripheral nerve blockade (PNB) is a particularly attractive technique because it is relatively easy to perform and it allows for low-risk, site-specific analgesia to the affected extremity [4]. Multiple studies have described the benefits of PNB in adult knee arthroscopy specifically, with benefits that include reduced hospital costs, decreased nausea and vomiting, shorter length of stay, decreased opioid utilization, and improved patient satisfaction [4-8]. A Cochrane review of randomized trials where adults received PNB combined with systemic analgesics (commonly opioids) or systemic analgesics alone surrounding major knee surgery found that the PNB combination group had significantly lower pain scores in the first 72 hours following surgery [9].

Increasingly, PNB is being employed as a component of the multimodal analgesic approach in pediatric orthopedic surgery. Prior to the development of ultrasound-guided techniques, PNB in pediatric patients using landmarks or nerve-stimulation methods was commonly considered infeasible due to concerns about the procedural risk, the need for deep sedation or general anesthesia during the procedure, which prevents active patient feedback during blockade, and the need to limit total local anesthetic volume to avoid toxicity, which could increase the risk of block failure [10-11]. Increasingly, investigators have demonstrated the feasibility of developing a regional anesthesia program for pediatric orthopedic surgery and the safety of using PNB in children [12-13]. A retrospective study comparing pediatric patients undergoing arthroscopic knee surgery with femoral nerve block versus patients receiving general anesthesia alone found that the patients with femoral nerve block showed a statistically significant reduction in hospital stay, decreased opioid requirements, and lower postoperative postanesthesia care unit (PACU) pain scores and those undergoing anterior cruciate ligament (ACL) repair had lower readmission rates [14]. However, postoperative pain in pediatric patients is still a significant challenge, and often the greatest challenge for families after pediatric patients have been discharged home, away from professional attention provider [15-16]. In addition, few studies have been carried out in adolescent patients specifically. Our study thus aimed to prospectively assess pain outcomes in pediatric patients after discharge from two archetypal sports procedures, arthroscopic ACL reconstruction, and/or meniscus surgery.

## Materials and methods

This was a single-center, single-surgeon prospective observational study conducted over a three-year period. After obtaining the Institutional Review Board approval, we approached and enrolled patients after obtaining written informed consent and assent from them. The inclusion criteria included: (1) age between 0 and 18 years old; (2) scheduled for arthroscopic knee surgery (ACL reconstruction and/or meniscus surgery); (3) scheduled for same day surgery; (4) case performed at ambulatory surgical center (ASC); and (5) anesthetic management including the use of single-shot PNB, femoral nerve blockade (FNB), and sciatic nerve blockade (SNB). All eligible patients were included. 

Anesthetic management

All patients and parents were consented for general anesthesia and PNB prior to induction of general anesthesia. Following induction of general anesthesia and airway management (laryngeal mask airway or endotracheal tube), the managing anesthesiologist performed a single-shot FNB and SNB (typically used for ACL reconstruction to cover posterior knee pain associated with hamstring autograft harvest) for postoperative analgesia. Single-shot FNB alone was utilized if SNB could not be placed easily, and the use of local anesthetic, block additives (clonidine), and ultrasound for PNB were at the discretion of the managing anesthesiologist. The patients were maintained under general anesthesia until the surgery concluded. Supplemental analgesics were given intraoperatively as per the anesthesiologist's discretion. Postoperatively, the patients recovered in the PACU with standard institutional protocols and all patients were discharged home on the day of surgery.

Surgical management

The primary procedure for patients in Group A was arthroscopic ACL repair (*n* = 18). In Group A, 11/18 (61.1%) patients underwent concomitant meniscus procedures, which included partial meniscectomy in 5/11 (45.5%), meniscus repair in 3/11 (27.3%), and combined meniscus repair and partial meniscectomy in 3/11 (27.3%). The primary procedure for patients in Group B was an arthroscopic meniscus procedure (n = 29), of which 15/29 (51.7%) underwent partial meniscectomy, 10/29 (34.5%) underwent meniscus repair, 3/29 (10.3%) underwent meniscectomy, and 1/29 (3.4%) underwent combined meniscus repair and partial meniscectomy.

Data collection

Data were collected using a telephone survey and chart review. Telephone surveys were conducted at 72 hours postoperatively. Patients or caregivers (depending on family preference) were asked to provide information on patient pain scores on postoperative day 3, day of sensation return to surgical extremity, frequency of opioid or NSAID use, and ability to complete activities of daily living (ADL) including ambulation, sleeping, and showering. Pain levels were assessed via the Numeric Rating Scale, a reliable, validated, and reproducible 11-point assessment where 0 refers to no pain and 10 refers to the worst possible pain, and respondents were asked to provide an integer value representing their pain [17]. Respondents were not compensated for completing the survey.

The chart review was performed to obtain perioperative data, which included demographic information, anesthesia and operative times, regional nerve block details, and occurrence of perioperative complications. Details on medications administered in the preoperative, intra-operative, and PACU were collected and reported. Results were analyzed using descriptive statistics as well as Pearson correlations via the SPSS version 24 software (IBM Corp. Released 2016. IBM SPSS Statistics for Mac, Version 24.0. Armonk, NY, USA). 

## Results

A total of 49 patients were enrolled but two were excluded because they could not be contacted at 72 hours to complete the postoperative survey. Thus, 47 patients were included for analysis (31/47 female, mean age 15.8 ± 2.1 years). Arthroscopic ACL reconstruction (Group A) was performed in 18 patients with 11/18 (61.1%) requiring concomitant meniscal surgery while arthroscopy with meniscus-only surgery (Group B) was performed in 29 patients. Demographic data (age, weight, gender, race) for each procedure group and surgical times are summarized in Table 1.  Although operative and total anesthesia times were substantially longer for Group A patients, time until anesthesia ready for procedure start was equivalent between procedure groups.

**Table 1:** Patient demographics and surgery details. Mean results are shown with standard deviations.

	Group A: ACL ± meniscus surgery	Group B: meniscus surgery
Mean age (years)	16.0 ± 1.4	15.7 ± 2.4
Mean weight (kilograms)	63.2 ± 9.6	63.5 ± 19.5
Sex [*n* (%)]		
*Male*	5 / 18 (27.8%)	11 / 29 (37.9%)
* Female*	13 / 18 (72.2%)	18 / 29 (62.1%)
Race [*n* (%)]		
*White*	11 / 18 (61.1%)	23 / 29 (79.3%)
*Black*	4 / 18 (22.2%)	4 / 29 (13.8%)
*Other*	3 / 18 (16.7%)	2 / 29 (6.9%)
Procedure laterality [*n* (%)]		
*Right*	5 / 18 (27.8%)	11 / 29 (37.9%)
* Left*	13 / 18 (72.2%)	18 / 29 (62.1%)
Mean anesthesia duration (minutes)	184 ± 27	89 ± 23
Mean operative duration (minutes)	137 ± 25	46 ± 23
Mean time until anesthesia ready (minutes)	21 ± 8	22 ± 7
Mean time for Block # 1 (minutes)	13 ± 7	13 ± 5
Mean time for Block # 2 (minutes)	17 ± 11	16 ± 6
Complications [*n* (%)]	0 / 18 (0%)	0 / 29 (0%)

Perioperative analgesic management, including peripheral nerve block (PNB) details are shown in Table 2. All patients received FNB with 0.2% ropivacaine (mean volume of 29 ml). There were 17/18 (94.4%) Group A patients who received SNB with ropivacaine (mean concentration 0.15%, mean volume 13 ml).  There were 25 / 29 (86.2%) Group B patients who received SNB containing ropivacaine (mean concentration 0.12%, mean volume 13 ml).

**Table 2:** Perioperative analgesic management. Mean results are shown with standard deviations. *Abbreviations*: PNB, peripheral nerve block; FNB, femoral nerve block; SNB, sciatic nerve block; PACU, post anesthesia care unit. Total dose amounts are adjusted based on patient weight in kilograms.

	Group A: ACL ± meniscus surgery	Group B: meniscus surgery
Femoral PNB (FNB) [*n* (%)]	18 / 18 (100%)	29 / 29 (100%)
FNB drug [*n* (%)]		
*Ropivacaine*	18 / 18 (100%)	29 / 29 (100%)
Mean FNB drug concentration (%)	0.20 ± 0.0	0.20 ± 0.0
Mean FNB drug volume (mililiters)	28.9 ± 7.9	28.9 ± 7.7
Patients receiving clonidine with FNB	10 / 18 (55.6%)	18 / 29 (62.1%)
Sciatic PNB (SNB) [*n* (%)]	17 / 18 (94.4%)	25 / 29 (86.2%)
SNB drug [*n* (%)]		
*Ropivacaine*	17 / 17 (100%)	25 / 25 (100%)
Mean SNB drug concentration (%)	0.15 ± 0.02	0.12 ± 0.03
Mean SNB drug volume (mililiters)	12.7 ± 4.4	12.9 ± 5.5
Premedication type [*n* (%)]		
*Midazolam*	1 / 18 (5.6%)	2 / 29 (6.9%)
*Acetaminophen*	16 / 18 (88.9%)	25 / 29 (86.2%)
Intraoperative analgesic type [*n* (%)]		
*Fentanyl*	8 / 18 (44.4%)	12 / 29 (41.4%)
*Ketorolac*	18 / 18 (100%)	29 / 29 (100%)
Intraoperative analgesic: mean total dose amount		
*Fentanyl (micrograms/kilogram)*	0.71 ± 0.49	0.89 ± 0.35
*Ketorolac (miligrams/kilogram)*	0.73 ± 0.26	0.63 ± 0.20
PACU analgesic type [*n* (%)]		
*Oxycodone*	9 / 18 (50%)	13 / 29 (44.8%)
*Fentanyl*	4 / 18 (22.2%)	5 / 29 (17.2%)
*Acetaminophen*	3 / 18 (16.7%)	1 / 29 (3.4%)
PACU analgesic: mean total dose amount		
*Oxycodone (miligrams/kilogram)*	0.087 ± 0.014	0.086 ± 0.017
*Fentanyl (micrograms/kilogram)*	0.50 ± 0.29	0.59 ± 0.30

Perioperative analgesic medication administration is summarized and shown in Table 3. At 72 hours postsurgery, there were no readmissions or complications related to pain. Median and interquartile ranges (IQR) of maximum PACU pain ratings were 0 (0.0-3.5) for Group A and 0 (0.0-2.5) for Group B. Median (IQR) for pain score at 72 hours postsurgery was 2.5 (1.0-5.0) and 5 (3.0-6.0) for Groups A and B, respectively. There were seven and nine patients in Groups A and B, respectively, who had maximum PACU pain ratings over zero. There were 17 and 26 patients in Groups A and B, respectively, who had 72-hour pain ratings over zero. Sensation to the affected limbs returned most commonly on the postoperative day one in both the groups (60% and 66%, respectively). Over 93% of all patients could ambulate and shower at 72 hours postoperatively. The analgesic usage patterns for the day of surgery (DOS), postoperative day one (POD1), postoperative day two (POD2), and postoperative day three (POD3) are shown in Table 3. At 72 hours postsurgery, approximately 96% of patients were still taking opioids and 98% were still taking an NSAID. Opioid doses decreased most significantly on postoperative day two; opioid usage appeared to decrease at a more rapid rate than NSAID usage. Approximately 22% and 21% experienced constipation with opioid usage from Groups A and B, respectively. 

**Table 3:** Postoperative follow-up outcomes. *Abbreviations*: PACU, post anesthesia care unit; NSAID, nonsteroidal anti-inflammatory drug.

	Group A: ACL ± meniscus surgery	Group B: meniscus surgery
PACU maximum pain score (mean ± SD, median, range)	1.8 ± 2.6, 0.0, (0.0-7.0)	1.7 ± 3.0, 0.0, (0.0-9.0)
72-Hour postoperative pain score (mean ± SD, median, range)	2.8 ± 1.9, 2.5, (0.0-7.0)	4.6 ± 2.2, 5.0, (0.0-8.0)
Day sensation returned [*n* (%)]		
* Day of surgery*	3 / 18 (16.7%)	4 / 29 (13.8%)
* Postoperative Day One*	12 / 18 (66.7%)	19 / 29 (65.5%)
*Postoperative Day Two*	3 / 18 (16.7%)	6 / 29 (20.7%)
*Postoperative Day Three*	0 / 18 (0%)	0 / 29 (0%)
Readmissions [*n* (%)]	0 / 18 (0%)	0 / 29 (0%)
Activities of daily living [*n* (%)]		
* Shower*	18 / 18 (100%)	26 / 29 (89.7%)
* Ambulation*	17 / 18 (94.4%)	28 / 29 (96.6%)
*Ambulation with crutches*	17 / 18 (94.4%)	26 / 29 (89.7%)
Using NSAID 72-hours postsurgery	18 / 18 (100%)	27 / 28 (96.4%)
NSAID type [*n* (%)]		
* Ibuprofen*	17 / 18 (94.4%)	28 / 29 (96.6%)
* Naproxen*	1 / 18 (5.6%)	0 / 29 (0%)
Prescription/over-the-counter [*n* (%)]		
*Prescription*	17 / 18 (94.4%)	27 / 29 (93.1%)
*Over-the-counter*	1 / 18 (5.6%)	2 / 29 (6.9%)
NSAID dose number (mean ± SD, median, range)		
*Day of surgery*	3.7 ± 0.7, 4.0, (2.0-4.0)	3.8 ± 0.6, 4.0, (2.0-4.0)
*Postoperative Day One*	3.7 ± 1.0, 4.0, (0.0-4.0)	3.8 ± 0.5, 4.0, (2.0-4.0)
Postoperative Day Two	3.6 ± 1.0, 4.0, (0.0-4.0)	3.8 ± 0.7, 4.0, (1.0-4.0)
*Postoperative Day Three*	3.2 ± 1.4, 4.0, (0.0-4.0)	3.3 ± 1.3, 4.0, (0.0-4.0)
NSAID-related side effects [*n* (%)]		
* Indigestion*	2 / 18 (11.1%)	1 / 29 (3.4%)
Using opioid 72-hours postsurgery	18 / 18 (100%)	27 / 29 (93.1%)
Opioid type [*n* (%)]		
*Oxycodone*	18 / 18 (100%)	26 / 29 (89.7%)
* Hydrocodone*	0 / 18 (0%)	1 / 29 (3.4%)
Number of opioid doses (mean ± SD, median, range)		
*Day of surgery*	4.8 ± 1.9, 6.0, (0.0-6.0)	5.1 ± 1.5, 6.0, (1.0-6.0)
* Postoperative Day One*	4.7 ± 2.1, 6.0, (0.0-6.0)	4.6 ± 2.0, 4.8, (0.0-6.9)
*Postoperative Day Two*	3.8 ± 2.3, 4.0, (0.0-6.0)	3.9 ± 2.2, 4.0, (0.0-6.9)
*Postoperative Day Three*	3.3 ± 2.6, 4.0, (0.0-6.0)	2.5 ± 2.6, 1.0, (0.0-6.9)
Opioid-related side effects [*n* (%)]		
* Constipation*	4 / 18 (22.2%)	6 / 29 (20.7%)
*Dizziness*	1 / 18 (5.6%)	2 / 29 (6.9%)
*Indigestion*	2 / 18 (11.1%)	2 / 29 (6.9%)
* Nausea/Vomiting*	3 / 18 (16.7%)	1 / 29 (3.4%)
* Itchiness*	1 / 18 (5.6%)	0 / 29 (0%)

To investigate whether the patients complaining of higher pain scores in the PACU had higher pain scores at 72 hours, we performed Pearson correlations. While we did not observe a statistically significant correlation in maximum pain scores and 72 hours pain scores for Group A (*r* = 0.418, p = 0.084), we did observe a positive correlation for Group B (*r* = 0.431, p = 0.025) that was statistically significant. 

## Discussion

This paper presents observational, short-term follow-up data for adolescents who received PNB for two common arthroscopic knee procedures. We did not observe any short-term complications or readmissions related to pain in our cohort. Additionally, we have described the recovery of these patients in the first 72 hours after discharge. It appears that these procedures are generally well tolerated with mild pain scores and early return of function status but use of opioid and NSAIDs remains common through postoperative day three. Sensation on the affected limb returned for the majority of patients (over 65%) on postoperative day one.

These are important findings given a previous study that suggests that pain in children may not be well controlled due to parental concerns about side effects [15]. In a 2010 longitudinal study of 132 parents of children aged 2-12 years undergoing elective outpatient surgery, Rony et al. compared preoperative parental attitudes about pain assessment and management with the children’s postoperative pain severity and analgesic administration for 48 hours postdischarge. The authors found that despite clinically significant levels of postoperative pain in the children enrolled in the study, the patients were only administered a median of one analgesic dose by their parents in 48-hours post-surgery. In that study, 73% of parents reported concerns of side effects and 52% were concerned of addiction [15]. It appears that in our cohort, pain was actively managed and side effects were minimal, and the use of PNB required extra counseling with parents regarding postoperative pain and sensation expectations.

Many other studies have reported similar findings of inadequate postdischarge analgesia in pediatric patients undergoing ambulatory orthopedic surgery [18-19], although the role of regional anesthesia is gaining popularity as a part of the multimodal anesthesia strategy to help mitigate postoperative pain. Regional nerve block may also facilitate more procedures to be completed at ambulatory surgery centers, which provide numerous benefits including estimated cost savings ranging from 17% to 43% depending on the procedure [20]. In our study, another compelling result was that the majority of patients in both the groups were able to shower and ambulate at 72-hours postsurgery. Earlier postoperative showering has been linked to greater patient satisfaction without any significant risk of surgical site infection [21-22].

While the femoral PNB has been shown to improve postoperative analgesia in adult arthroscopic ACL repair [23-25], some patients with femoral-only PNB complained of postoperative posterior knee pain. A combined femoral and sciatic nerve PNB has been shown to improve postoperative analgesia compared to femoral PNB alone [26]. Risks of PNB that include vascular puncture and bleeding, nerve damage, and local anesthetic systemic toxicity (LAST) are rare, and single-injection PNB has not been associated with infection [4, 27]. Most patients in our study received both femoral and sciatic PNBs, and the few who received femoral PNB only were due to procedural difficulty. The time periods to complete PNBs were short, with all averages under 20 minutes (Table 1).

Our patients also demonstrated a clinically minor level of average postoperative pain, as shown by studies where ratings between 1 and 4 are considered mild [17, 28]. In addition, the interquartile ranges appear generally insignificant with changes of approximately 3 on a 11-point scale considered clinically significant [29]. The small numbers of patients with maximum PACU pain ratings over zero may represent incomplete blocks or blocks outside the surgical area; however, the use of maximum values may not represent the average pain experience of patients. Interestingly, meniscus-only procedures were reported to be more painful in our cohort, an area of potential future inquiry, but this difference was statistically insignificant (p = 0.6). It is clear from the pain score data that the PNB did work as expected to reduce the immediate postoperative pain to minimal (median 0 in PACU for both surgery types). However, the median 72-hour pain rating’s increase to 2.5 and 5.0 for the ACL and meniscal groups, respectively, shows that when the block wore off, pain worsened. Pain ratings of patients between hospital discharge and 72 hours postoperatively were not obtained. 

Daily opioid and NSAID analgesic doses required by patients in the first 72 hours postoperatively are summarized and shown in Table 3. Of note, analgesic requirements decrease the greatest on the postoperative day two for both procedure groups and analgesic types. Postoperative opioid usage appears slightly greater in the ACL group, however, as a larger procedure, it is not unexpected that these patients would require more opioids postoperatively. Furthermore, the level of opioid use was relatively high for both patient groups overall, and it would be interesting to study in the future whether patients using nonopioids alone would indicate similar pain relief as those continuing to use opioids. Overall, our goal is to find strategies that will continue to minimize the need for opioid usage.

Most patients’ sensation to the affected limb returned on postoperative day one, consistent with the literature which suggests that PNBs have 12-24 hours duration of action [4]. The statistically significant correlation of median maximum PACU pain scores and 72-hour postoperative pain scores in Group B suggest that patients reporting high levels of pain early postoperatively tend to report high levels of pain later postoperatively also. Although this correlation did not reach statistical significance in Group A, this same trend appears to be evident in this group as well. Greater research is required to examine potential reasons for higher analgesic needs in certain patients.

As far as limitations and future directions of this work, our study is most significantly limited by the sample size. Furthermore, future studies would benefit from a direct comparison to a control group that did not receive PNB, which is lacking in this study although it was designed as an observational study. Timepoints in addition to 72 hours could be included to generate normative data on pain levels throughout the postoperative period. Other limitations include a lack of standardized anesthesiologist and a lack of long-term follow-up; however, low PACU pain scores suggest all patients had functioning blocks. The latter point is particularly important as recent research has suggested that pediatric and adolescent patients who underwent ACL reconstruction with PNB had decreased quadriceps strength at six months postoperatively compared to patients who did not receive PNB [30]. In this study, muscle weakness was not specifically assessed; however, a challenge of this assessment is directly attributing muscle weakness to PNBs versus other factors like incision-site pain or atrophy from the injury. More detailed descriptions of limb sensation return could be included. Future studies could also be expanded to include additional procedure types or different peripheral nerve block concentrations and volumes. For example, Veneziano et al. reported that ropivacaine 0.5% offers superior analgesia compared to ropivacaine 0.2% and bupivacaine 0.25% by resulting in quicker hospital/PACU discharge and lesser opioid requirements postoperatively after pediatric knee arthroscopies [11].  

## Conclusions

Regional nerve block appears to be an effective and safe analgesic strategy for pediatric arthroscopic ACL and meniscus procedures, with no short-term complications or readmissions related to pain in our cohort. Future prospective investigation is needed to characterize long-term pain outcomes in this surgical population, and our study is limited by the sample size and the lack of a randomized control group. However, our study uniquely offers a snapshot of the postoperative pain levels, analgesic needs, and ADL of pediatric patients for 72 hours after discharge from ambulatory knee arthroscopy and affirms the efficacy and safety of PNBs with no readmissions or complications and overall low levels of postoperative pain, which increase after PNB effects dissipate.
